# Small Molecule CCR4 Antagonists Protect Mice from *Aspergillus* Infection and Allergy

**DOI:** 10.3390/biom11030351

**Published:** 2021-02-25

**Authors:** Silvia Bozza, Rossana Giulietta Iannitti, Marilena Pariano, Giorgia Renga, Claudio Costantini, Luigina Romani, Jagadeesh Bayry

**Affiliations:** 1 Department of Medicine and Surgery, University of Perugia, 06132 Perugia, Italy; silvia.bozza@unipg.it (S.B.); r.iannitti@srfarmaceutici.com (R.G.I.); marilena.pariano@gmail.com (M.P.); rengagiorgia@gmail.com (G.R.); costacla76@gmail.com (C.C.); 2 Institut National de la Santé et de la Recherche Médicale, Centre de Recherche des Cordeliers, Sorbonne Université, 75006 Paris, France

**Keywords:** CCR4, *Aspergillus fumigatus*, vaccine, cystic fibrosis, invasive aspergillosis, allergic bronchopulmonary aspergillosis

## Abstract

The ability to regulate the recruitment of immune cells makes chemokines and their receptors attractive drug targets in many inflammatory diseases. Based on its preferential expression on T helper type 2 (Th2) cells, C-C chemokine receptor type 4 (CCR4) has been widely studied in the context of allergic diseases, but recent evidence on the expression of CCR4 in other cell types has considerably expanded the potential applications of CCR4 antagonism. However, the current number of approved indications, as well as the portfolio of CCR4-targeting drugs, are still limited. In the present study, we have assessed the potential therapeutic efficacy of a CCR4 small molecule antagonist, SP50, discovered via an in silico-based approach, against a variety of pre-clinical settings of infection with the fungus *Aspergillus fumigatus*. We show that SP50 efficiently worked as prophylactic vaccine adjuvant in immunocompetent mice, protected against invasive aspergillosis in immunosuppressed mice. Further, the CCR4 antagonist prevented allergic bronchopulmonary aspergillosis in susceptible mice, and in a murine model of cystic fibrosis, a genetic disorder characterized by chronic pulmonary inflammation and recurrent infections. In conclusion, our results extend the potential applications of CCR4 antagonism and prompt for the development of novel compounds with the potential to progress to clinical trials.

## 1. Introduction

Chemokines are cytokines endowed with chemotactic activity and play critical roles in the positioning and migration of immune cells in homeostasis and during immune responses [1]. Approximately 50 chemokines and 20 chemokine receptors, along with 5 atypical receptors, have been described so far [2]. Although a receptor–ligand promiscuity exists, the individual chemokine receptor profile and the context-dependent production of chemokines, among other mechanisms, may coordinate the selective recruitment of populations of immune cells [3]. Since chemokines are dysregulated in many inflammatory diseases, pharmacological inhibition of the relevant chemokine receptors represents an active area of research. However, the knowledge of the chemokine/chemokine receptor system is a fundamental prerequisite for successful pharmacological targeting [4]. 

Among chemokine receptors, CCR4 has attracted considerable interest as a potential target in a variety of pathological conditions [5,6]. CCR4 is the chemokine receptor preferentially expressed on Th2 cells. However, other T cell subsets can express CCR4, including cutaneous lymphocyte antigen (CLA)-positive skin-homing T cells, regulatory T cells (Treg cells), Th17, and Th22 cells [7]. In addition, CCR4 was found to be expressed by natural killer (NK) cells, NKT cells, eosinophils, platelets, and basophils [7]. CCR4 has two known ligands, CCL17 and CCL22, also known as thymus- and activation-regulated chemokine (TARC) and macrophage-derived chemokine (MDC), respectively, that differ in receptor binding properties, expression profile, and modulatory activity of immune functions [8]. The predominant expression of CCR4 in Th2 cells, as well as the upregulation of CCL17 and CCL22 in allergic diseases, have captured attention for their potential therapeutic targeting of CCR4 in the treatment of allergies [5]. Similarly, the expression of CCR4 in skin-homing CLA+ T cells has attracted interest for use in atopic dermatitis therapy [5]. Another area of research is represented by cancer immunotherapy. Indeed, CCR4 is expressed by Treg cells whose accumulation in the tumor microenvironment is a cause of resistance to immune therapy. CCR4 antagonism may therefore prevent Treg tumor infiltration and increase the antitumoral response [9]. Therefore, CCR4 is increasingly being identified in different pathologies, thus making the development of effective CCR4-targeting drugs a potential therapeutic strategy with wide-ranging applications. 

Several classes of drugs targeting CCR4 have been developed, including small molecule antagonists and biologicals. The former include class I and class II molecules that bind the extracellular or the intracellular portion of the receptor, respectively, and include lipophilic heteroarenes and aryl sulphonamides [10], with only one compound progressing to clinical trials so far. The latter include mogamulizumab, a fully humanized monoclonal antibody [6], approved by the U. S. Food and Drug Administration (FDA) for the treatment of two rare types of non-Hodgkin lymphoma. Therefore, the current portfolio of CCR4-targeting drugs as well as the number of approved indications is limited, thus making the exploration of novel clinical scenarios and drug candidates an open field of investigation.

*Aspergillus fumigatus* is one of the highly abundant airborne fungal species. *Aspergillus* species cause significant morbidity and mortality in the population by eliciting a wide spectrum of diseases that depend not only on the degree of exposure, but also on the characteristics of the immune response [11]. Clinically, the symptomatology could range from severe and life-threatening forms of invasive aspergillosis in immunocompromised individuals to allergic asthma in immunocompetent population [12]. *A. fumigatus* is responsible for an extreme form of allergic bronchopulmonary aspergillosis (ABPA), characterized by an exaggerated hypersensitivity reaction [13]. Allergic asthma is typically driven by Th2 cells that predominantly express CCR4. Significantly enhanced Aspergillus-specific IgE and eosinophilia are the characteristic features of ABPA. Moreover, *A. fumigatus* is by far the most common fungal species isolated in the sputum of cystic fibrosis (CF) patients [14,15]. CF is a rare genetic disorder caused by mutations in the gene encoding for the ion channel Cystic Fibrosis Trans-membrane conductance Regulator (CFTR), involved in the regulation of the ionic content of luminal fluid [16]. A deterioration of the respiratory functions by a chronic inflammatory state and recurrent infections are the main causes of morbidity and mortality in CF patients [17]. Pulmonary exacerbations are caused by bacteria, such as *Pseudomonas aeruginosa* and *Staphylococcus aureus*, but also *A. fumigatus*.

Though the survival rate with invasive aspergillosis has been improved recently due to the effective use of antifungal agents, incidence of drug resistance is growing. Also, the use of steroids in allergic diseases is associated with steroid resistance and immunosuppression. In view of these facts, the current research on the therapy of *A. fumigatus*-associated pathologies is focused on immunomodulation and vaccines. Therefore, in the present manuscript, we have extended the potential clinical application of CCR4 antagonism in different pre-clinical models of *Aspergillus* infection and allergy. Our results indicate that CCR4 antagonists can protect from aspergillosis and allergic bronchopulmonary aspergillosis (ABPA) in susceptible mice and in a murine model of cystic fibrosis (CF), thus extending the clinical indications for therapeutic development of CCR4 targeting drugs.

## 2. Materials and Methods

### 2.1. Mice

Murine experiments were performed according to the Italian Approved Animal Welfare Authorizations 360/2015-PR (date of approval 15 May 2015) lasting for five years (2015–2020) and Legislative decree 26/2014 regarding the animal license, obtained by the Italian Ministry of Health. Four- to six-week-old C57BL/6 mice were purchased from Charles River (Calco, Italy). Breeding pairs of CF mice homozygous for the F508del-CFTR that had been backcrossed for 12 generations to the C57BL/6 strain, or in the FVB/129 outbred background (*Cftrtm^1EUR^*, F508del, abbreviated *Cftr^F508del^* mice), were obtained from Bob Scholte, Erasmus Medical Center Rotterdam, The Netherlands [18] and maintained in our animal facility. Mice were treated as described below and monitored for fungal growth, expressed as log10 CFU per organ, mean ± SD. Bronchoalveolar lavage (BAL) was performed by cannulating the trachea and washing the airways with 3 × 0.5 mL of PBS to collect the BAL fluid. Total and differential cell counts were done by staining BAL smears from allergic mice with May-Grünwald Giemsa reagents (Sigma-Aldrich, St. Louis, MO, USA) before analysis.

### 2.2. Vaccination Model

The vaccination model consisted in administering mice with the *Aspergillus* Cat1p antigen along with the immunoadjuvant murine CpG oligodeoxynucleotide 1862 (CpG/Ag model), as described [19]. Briefly, mice were injected intranasally (i.n.) with 2 × 10^7^ *Aspergillus* conidia/20 μL of saline (see below) 14 days after treatment with 5 μg of Cat1p + CpG 10 nM/20 μL of saline, with and without 5 µg/20μL/i.n. SP50 (AF-399/42016530; C_26_H_17_N_3_O_4_S_3_; 4-(1-benzofuran-2-ylcarbonyl)-1-[5-(benzylsulfanyl)-1,3,4-thiadiazol-2-yl]-3-hydroxy-5-(2-thienyl)-1,5-dihydro-2H-pyrrol-2-one) [20,21], administered 14, 7, and 3 days before. Mice were immunosuppressed with 150 mg/kg cyclophosphamide by intraperitoneal injection (i.p.) a day before infection.

### 2.3. Aspergillus Infection and Allergy

Viable conidia (95%) of *A. fumigatus* (Af293) were obtained by growth on Sabouraud dextrose agar (Sigma-Aldrich) supplemented with chloramphenicol for 5 days at room temperature. The fungus was collected with a cell scraper after addition of PBS/0.05% Tween 80, transferred in a tube, pelleted and resuspended in PBS. After counting, the resuspension was further diluted in PBS to reach the desired final concentration. For in vivo experiments, mice were anesthetized by intraperitoneal injection of 2.5% avertin (Sigma Chemical Co, St. Louis, MO, USA) before intranasal instillation of a suspension of 2 × 10^7^ resting conidia /20 μL saline. For allergy, mice received an i.p. and a subcutaneous injection of 100 μg of *A. fumigatus* culture filtrate extract (CCFA) dissolved in Freund’s incomplete adjuvant (Sigma-Aldrich) followed by two consecutive i.n. injections (1 wk apart) of 20 μg of CCFA [22]. Mice were treated with SP50 (5 µg/20μL/i.n.) daily 2 h after the *Aspergillus* infection (infection model) or twice, in concomitance with the first and second administration of CCFA (allergy model). Control mice received the same volume of vehicle PBS. Lungs were removed and immediately fixed in 10% neutral buffered formalin (Bio-Optica Milano Spa, Milan, Italy) for 24 h, embedded in paraffin, sectioned into 3–4 μm and stained with Periodic Acid-Schiff reagent. Histology images were acquired using a microscope (BX51) with a 40× objective equipped with a high-resolution DP71 camera (Olympus, Tokyo, Japan).

### 2.4. Immunofluorescence

The tissues were removed and fixed in 10% phosphate-buffered formalin (Bio-Optica Milano Spa, Milan, Italy), embedded in paraffin and sectioned at 3 µm. Sections were rehydrated and, after antigen retrieval in citrate buffer (10 mM, pH6), were fixed in 2% formaldehyde for 40 min at room temperature and permeabilized in a blocking buffer containing 5% FBS, 3% BSA, and 0.5% Triton X-100 in PBS. The slides were then incubated at 4 °C with primary antibody anti-IL-4 (eBioscience, San Diego, CA, USA), anti-IL-10 (eBioscience) or anti-forkhead box protein P3 (FOXP3; Abcam, Cambridge, UK). After extensive washing with PBS, the slides were then incubated at room temperature for 60 min with secondary antibody goat anti-Rabbit TRITC (Bethyl Laboratories, Inc., Montgomery, TX, USA). Nuclei were counterstained with 4′,6-diamidino-2-phenylindole (DAPI). Images were acquired using a microscope BX51 and analySIS image processing software (Olympus).

### 2.5. ELISA

Murine IL-4, IL-5, IL-10, IL-13, IFN-γ, IL-17A levels were determined in organ homogenates by using specific ELISA according to manufacturers’ instructions (R&D Systems, Minneapolis, MN, USA).

### 2.6. RT-PCR

Real-time PCR was performed using the CFX96 Touch Real-Time PCR detection system and iTaq™ Universal SYBR^®^ Green Supermix (BioRad, Hercules, CA, USA). Organs were lysed and total RNA was extracted using TRizol™ Reagent (Thermo Fisher Scientific, Waltham, MA, USA) and reverse transcribed with PrimeScript RT Reagent Kit with gDNA Eraser (Takara Bio, Otsu, Japan), according to the manufacturer’s directions. Amplification efficiencies were validated and normalized against β-actin. The thermal profile for SYBR Green real-time PCR was at 95 °C for 3 min, followed by 40 cycles of denaturation for 30 s at 95 °C and an annealing/extension step of 30 s. Each data point was examined for integrity by analysis of the amplification plot. The mRNA-normalized data were expressed as relative mRNA levels with respect to control. The following murine primers were used: *β-actin*: forward AGCCATGTACGTAGCCATCC, reverse CTCTCAGCTGTGGTGGTGAA; *Ifng*: forward ACTGGCAAAAGGATGGTGAC, reverse TGAGCTCATTGAATGCTTGG; *Il10*: forward GAGAAGCATGGCCCAGAAATCAAG, reverse ATCACTCTTCACCTGCTCCACTGC; *Il17a*: forward GACTACCTCAACCGTTCCAC, reverse CCTCCGCATTGACACAGC; *Il17f*: forward CCCTGGAGGATAACACTGTGAGAG, reverse TTCCTGACCCTGGGCATTGATG; *Il22*: forward CTGCCTGCTTCTCATTGCCCTGTG, reverse GATGTACGGCTGCTGGAAGTTGG; *Il4*: forward CGGCATTTTGAACGAGGTCACAGG, reverse AGCACCTTGGAAGCCCTACAGACG; *Muc1*: forward TGAGCCAGGACTTCTGGTAG, reverse CCTTCTGAGAGCCACCACTA; *Muc5ac*: forward CTGGACCTGGAGGTTGTATG, reverse CAGTAGTGAGGGTTGGATGG; *Muc13*: forward ACATGGTGAAGGGTCAAGAA, reverse AGATGAACTACCCACGGTCA.

### 2.7. Statistical Analysis

GraphPad Prism software 6.01 (GraphPad, San Diego, CA, USA) was used for the analysis. Data are expressed as mean ± SD. Statistical significance was calculated by one-way ANOVA (Tukey’s or Bonferroni’s post hoc test) for multiple comparisons and by a two-tailed Student’s t-test for single comparison. We considered all *p* values < 0.05 significant. The in vivo groups consisted of 3–5 mice/group.

## 3. Results

### 3.1. The CCR4 Antagonist SP50 Promotes Vaccine-Induced Resistance

**Figure 1:**
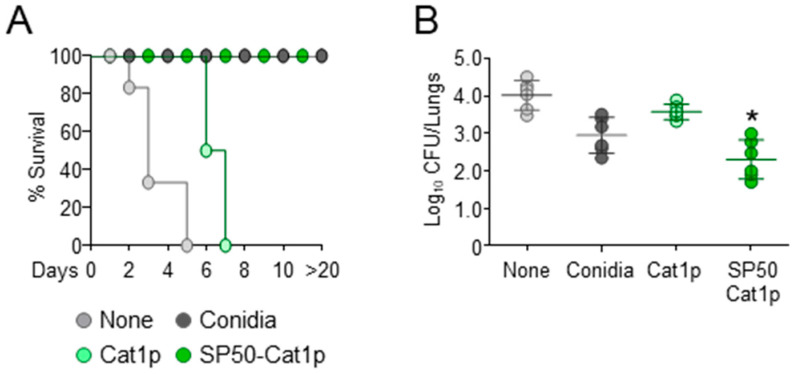
Vaccinating potential of SP50 against invasive pulmonary aspergillosis. C57BL/6 mice were injected intranasally with 2 × 10^7^ *Aspergillus* conidia 14 days before (Conidia) or with Ag (Cat1p), with and without SP50, and CpG (10 nM) 14, 7, and 3 days before the intranasal infection with 2 × 10^7^ live, resting conidia. Mice were immunosuppressed i.p. with 150 mg/kg cyclophosphamide a day before infection. Mice were assessed for (**A**) survival, and for (**B**) fungal growth (Log10 CFU) in the lungs 3 days post-infection. Data are pooled from three independent experiments. None, infected mice. * *p* < 0.05, SP50-Cat1p-treated vs Cat1p-treated mice.

By using a combination of in silico and in vitro screening-driven approaches, Bayry et al. described the development of small molecule CCR4 antagonists that were able to target Treg cells and work as vaccine adjuvant by boosting immune responses when used in combination with either Modified Vaccinia Ankara expressing Ag85A from *Mycobacterium tuberculosis* or recombinant hepatitis B virus surface antigen vaccines [20]. We have previously evaluated the ability of *A. fumigatus* proteins, glycolipids, and polysaccharides to induce vaccine-dependent protection in mice [19]. Among the different antigens, the mycelia catalase 1 (Cat1p) was unable to confer protection [19]. Therefore, by resorting to the CpG/Ag model, we asked whether the vaccination potential of Cat1p against pulmonary aspergillosis could be improved by the CCR4 antagonist [19]. In agreement with previous findings, vaccination with *A. fumigatus* conidia protected mice from subsequent infection with conidia while mice rapidly succumbed when Cat1p was used (Figure 1). However, concomitant treatment with the CCR4 antagonist SP50 [20,21] significantly reduced fungal burden and conferred full protection against infection (Figure 1), thus confirming the vaccine adjuvant properties of CCR4 antagonists.

### 3.2. The CCR4 Antagonist SP50 Protects Immunosuppressed Mice from Invasive Aspergillosis

**Figure 2:**
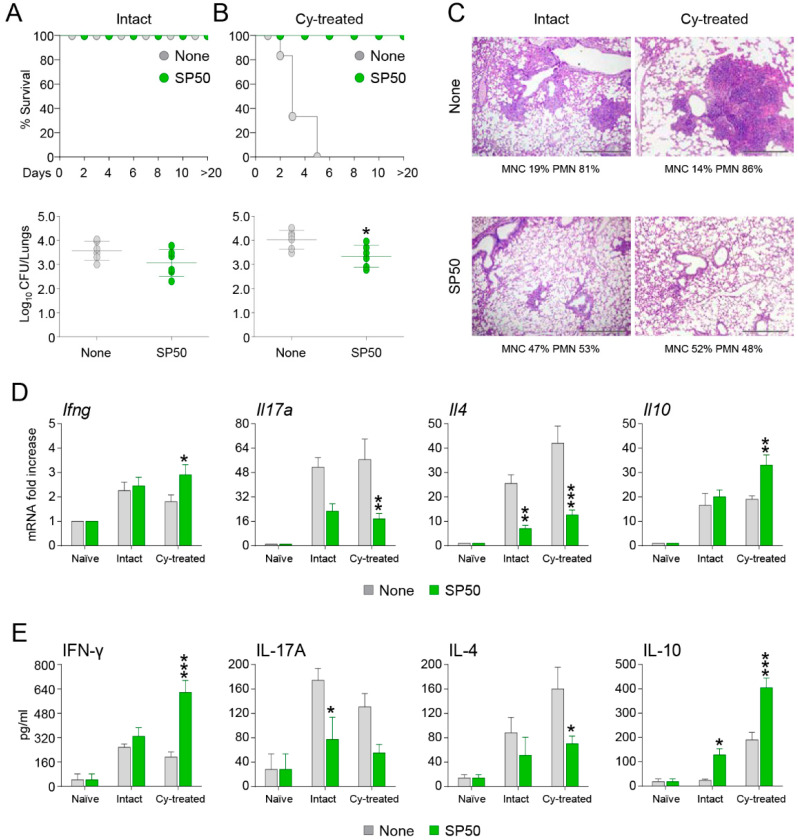
SP50 protects mice from lethal intranasal *A. fumigatus* infection. Intact (**A**) or (**B**) cyclophosphamide (Cy)-treated mice (150 mg/kg a day before infection) received intranasally 2 × 10^7^ *A. fumigatus* conidia. Mice were treated with SP50 (5 µg/mouse/i.n.) in concomitance with *Aspergillus* infection. Mice were assessed for survival, and for fungal growth (Log10 CFU) in the lungs at 3 days post-infection. In (**C**) lung histology (periodic acid-Schiff staining) of *A. fumigatus*-infected and SP50-treated mice is shown. Values represent percentages of polymorphonuclear (PMN) or mononuclear (MNC) cells in the BAL fluid. (**D**) Gene expression analysis for *Ifng*, *Il17a*, *Il4*, and *Il10* in lungs was done by RT-PCR 3 days after the infection. (**E**) Cytokines were determined in lung homogenates 3 days after the infection by ELISA. Data are pooled from three independent experiments. *, *p* < 0.05, **, *p* < 0.01, ***, *p* < 0.001, SP50-treated vs SP50-untreated (None) mice.

We then analyzed the effects of the CCR4 antagonist in other models of aspergillosis. Invasive aspergillosis is an important cause of morbidity and mortality in immunocompromised patients [23]. Hence, we first resorted to cyclophosphamide immunosuppressed mice and evaluated whether the CCR4 antagonist SP50 could protect mice from lethal i.n. *A. fumigatus* infection. As shown in Figure 2, while cyclophosphamide-treated mice rapidly succumbed to infection, the concomitant administration of SP50 completely protected mice from death. The increased survival was associated with a reduced fungal burden (Figure 2B), improved histopathology (Figure 2C), and reduced levels of IL-17A and IL-4 mRNA and protein, while IFN-γ and IL-10 levels were significantly increased (Figure 2D,E). Interestingly, the same changes in fungal burden, histopathology and cytokine profile were also observed in untreated mice (Figure 2A–E), indicating that the CCR4 antagonist is effective against the immune response elicited by *Aspergillus* infection.

### 3.3. The CCR4 Antagonist SP50 Protects Mice from ABPA

**Figure 3:**
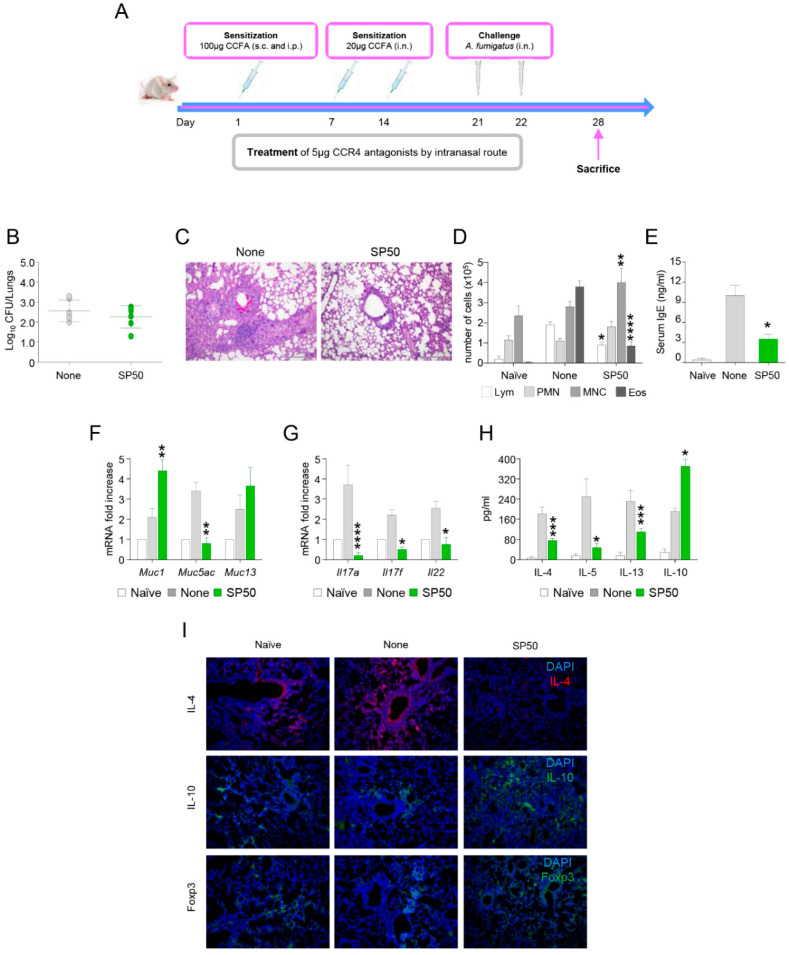
SP50 protects mice from allergic bronchopulmonary aspergillosis. (**A**) Representation of the murine experimental model of allergy. C57BL/6 mice was sensitized by the concomitant intraperitoneal (100 μg) and subcutaneous (100 μg) administration of *A. fumigatus* culture filtrate extract followed 1 week later by the intranasal instillation of 20 μg of the extract. After an additional 7 days, bronchial colonization of *A. fumigatus* was induced by resting conidia administered i.n. (2 × 10^7^), and the animals were evaluated 1 week later for parameters of allergic airway inflammation. Treatments included SP50 administered at 5 μg/mouse/i.n. twice, in concomitance with the first and second CCFA administrations. (**B**) Mice were assessed for fungal growth (Log10 CFU) in the lungs at 1 week post-infection. (**C**) Paraffin-embedded lung sections were stained with periodic acid-Schiff to visualize mucin, goblet cells, and inflammatory cells. (**D**) The abundance of eosinophils (Eos), monocytes (MNC), polymorphonucleates (PMN), and lymphocytes (Lym) was assessed in BAL fluid. (**E**) IgE antibodies were determined in sera. (**F**,**G**) Gene expression analysis in lungs was done by RT-PCR a week after the infection. (**H**) Cytokines were determined in lung homogenates 7 days after the infection by ELISA. (**I**) Immunofluorescence staining with anti-IL-4, anti-IL-10, and anti-Foxp3 antibody of lungs at 7 dpi. Cell nuclei were stained blue with DAPI. Representative images were acquired with a high-resolution Microscopy Olympus DP71 with a × 20 objective. Data are pooled from three independent experiments. Control, naïve C57BL/6 mice. *, *p* < 0.05, **, *p* < 0.01, ***, *p* < 0.001, ****, *p* < 0.0001, SP50-treated vs SP50-untreated (None) mice.

Another important cause of morbidity by *A. fumigatus* is represented by allergic asthma. As mentioned above, the pathogenesis of allergic asthma is driven by CCR4-expressing Th2 cells. Based on this premises, we resorted to a murine model of ABPA (Figure 3A) in the presence or absence of the CCR4 antagonist SP50. As shown in Figure 3, while the administration of SP50 did not significantly decrease fungal burden (Figure 3B), the lung histopathology was dramatically improved (Figure 3C). In line with these findings, SP50 significantly reduced the recruitment of eosinophils (Figure 3D) and serum IgE levels (Figure 3E). Moreover, the levels of the mucin MUC5AC, known to be upregulated in allergic inflammatory responses [24], were reduced by SP50 while the MUC1 and MUC13 were increased (Figure 3F). Finally, consistent with an inhibitory activity of the CCR4 antagonists against Th2 cells, the levels of IL-4, IL-5, and IL-13 were all reduced by SP50 (Figure 3H,I). Interestingly, also the levels of Th17 cytokines IL-17A, IL-17F, and IL-22 were reduced by SP50, indicating an inhibition of Th17 cells (Figure 3G). By contrast, the levels of IL-10 were increased (Figure 3H,I).

In addition, FoxP3^+^ cells were also enhanced in SP50 treated mice (Figure 3I).

Collectively, these results reinforce the potential application of CCR4 antagonists against allergic asthma. In this report, we extended our investigation to allergy model induced by *A. fumigatus* and showed that CCR4 antagonists render protection by targeting Th2 cells.

### 3.4. The CCR4 Antagonist SP50 Protects a Murine Model of Cystic Fibrosis from Aspergillosis

**Figure 4:**
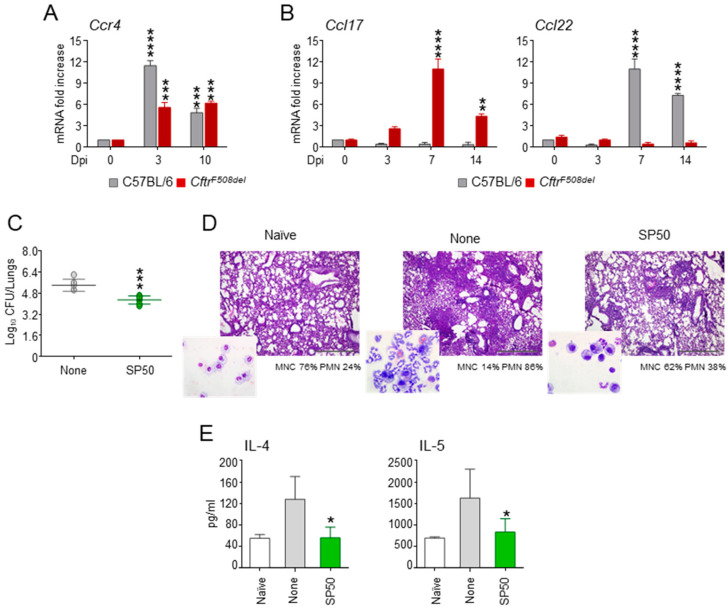
SP50 protects from aspergillosis in a murine model of cystic fibrosis. C57BL/6 and *Cftr^F508del^* mice were infected intranasally (in) with *A. fumigatus* and treated with SP50 administered (5µg/mouse/in) in concomitance with *Aspergillus* inoculation. (**A**) Expression of CCR4 and (**B**) its ligands CCL17 and CCL22 during infection. (**C**) *Cftr^F508de^*^l^ mice were assessed for fungal growth (Log10 CFU) in the lungs at 7 days post-infection (Dpi). In (**D**) lung histology (periodic acid-Schiff staining) of *A. fumigatus*-infected and SP50-treated mice (% of neutrophils in the bronchoalveolar lavage are shown in the insets). (**E**) Cytokines were determined in lung homogenates 7 days after the infection by ELISA. Data are pooled from three independent experiments. Naïve, uninfected and untreated mice. *, *p* < 0.05, **, *p* < 0.01, ***, *p* < 0.001, ****, *p* < 0.0001, SP50-treated treated vs untreated (None) mice and Dpi 3 and 10 vs 0.

Based on previous results, we resorted to a murine model of CF challenged with *A. fumigatus* and first evaluated the expression of CCR4 and its ligands CCL17 and CCL22 during infection. Both wild-type and CF mice were characterized by an increase in the levels of CCR4 (Figure 4A) while CCR4 ligands displayed a strain-selective behaviour, with CCL17 and CCL22 increasing in CF and wild-type mice, respectively (Figure 4B). This finding is particularly interesting because CCL17 and CCL22 differ in their ligand binding properties. Indeed, as opposed to CCL17, CCL22 potently and rapidly induce CCR4 internalization [25]. This would suggest that in CF lungs the chemokine/chemokine receptor system is kept functional for prolonged times with potential pathogenic effects. This is in agreement with CCL17 and CCR4 likely playing a critical role in ABPA in CF [26]. We then evaluated the effect of SP50 in *A. fumigatus*-induced pathology. As shown in Figure 4, SP50 reduced fungal burden (Figure 4C), ameliorated lung histopathology (Figure 4D), and reduced neutrophil infiltration (in the insets of Figure 4D). The levels of the Th2 cytokines IL-4 and IL-5 were reduced by SP50 (Figure 4E).

These data indicate that CCR4 antagonists may represent a valuable strategy to treat *A. fumigatus* infection in CF patients by reducing the allergic inflammatory response that occurs in the lungs of these patients.

## 4. Discussion

The results presented in this manuscript expand the potential application of small molecule CCR4 antagonists to include *Aspergillus* infection in various clinical scenarios, ranging from invasive aspergillosis in susceptible host to allergic disorders upon sensitization. In addition, CCR4 antagonists may be used to increase the protective immune response to vaccination with fungal antigens and to confer protection against subsequent infection. The ability of CCR4 antagonists to protect against *Aspergillus*-induced allergic diseases did not come without expectation. Indeed, based on its predominant expression in Th2 lymphocytes, CCR4 has long been considered as candidate target for drug development against allergy and asthma. Indeed, Schuh et al. have demonstrated that CCR4-deficient mice display a lower airway hyperresponsiveness to *A. fumigatus* in sensitized mice at later times after conidia challenge, with more efficient clearance of conidia; reduction in eosinophils; and reduced levels of IL-4, IL-5, and IL-13 [27]. We could confirm the reduction of eosinophil infiltration and IgE serum level in the presence of a CCR4 antagonist. However, while the study of Schuh et al. did not detect any changes in airway remodeling in the presence or absence of CCR4, with similar peribronchial fibrosis and goblet cell hyperplasia [27], we found a reduction in the levels of the mucin MUC5AC, likely corresponding to an effect towards the resolution of the allergic response. In line with this result, we could also unravel an effect of the CCR4 antagonist on Th17 cells, which contribute to airway remodeling in chronic allergic airway inflammation [28]. IL-17 is balanced by IL-10 that works to promote the resolution of inflammation induced by continuous antigen stimulation. Consistently, our data indicate that reduction of IL-17 by CCR4 antagonists is paralleled by an increase in IL-10 levels, thus indicating that inhibition of CCR4 not only prevents Th2 recruitment, but also shift the balance towards a tolerogenic state. Similar results were obtained in a murine model of CF that we have previously demonstrated to be more susceptible to *Aspergillus* infection and allergy, likely because of a dysregulated Th17/Treg balance, in turn linked to a defective indoleamine-2,3-dioxygenase activity [29]. In the present study, we demonstrate that the CCR4 antagonist promotes tolerance in CF mice, by increasing the levels of IL-10 while reducing IL-4, IL-5, and IL-17. We believe that IL-10 could be contributed from locally differentiated Treg and/or Tr1 cells. As Th17 response and cytokines are reduced in CCR4 antagonists-treated mice, and that Th17 and Treg responses are reciprocally regulated, it might tilt the balance towards induced Tregs. In fact, immunofluorescence staining has shown increased Foxp3^+^ cells in the lungs of ABPA mice treated with CCR4 antagonist. The identification of source of IL-10 is a subject of future investigation.

The ability of CCR4 antagonists to protect against invasive aspergillosis was less expected. A previous study in neutrophil-depleted mice demonstrated that the relative absence of CCR4 protected mice against invasive aspergillosis likely by preventing the immunosuppressive activities of CCL17 [30]. We used a different model of immunosuppression based on the chemotherapeutic drug cyclophosphamide and confirmed that CCR4 inhibition protected mice from invasive aspergillosis. Similar to the allergy model, CCR4 inhibition resulted in a reduction of Th2/Th17-associated cytokines, while the levels of IL-10 were increased. In addition, we also found an increase in the levels of the Th1 cytokine IFN-γ. This is in line with the previous study in which neutrophil-depleted mice had increased levels of IL-12 when deficient in CCR4 [30], which may be correlated with the increased levels of IFN-γ that we observe. Currently, the source of IFN-γ in CCR4 antagonist-treated mice either in *Aspergillus* infection or allergy models is not known. We have previously shown that vaccination with CCR4 antagonists also enhances CD8+ cytotoxic T cell (CTL) responses to the tumor antigens [31]. Whether vaccination with CCR4 antagonists in *Aspergillus* infection or allergy models also induces CTL responses remain to be determined.

Besides identifying the clinical indications for the application of CCR4 antagonists, it is important to develop drugs that can progress to clinical trials and be used in patients. In this manuscript, we have used the compound SP50 [20]. The key findings were confirmed with novel peptide-based CCR4 antagonists synthesized by a fluorenylmethoxycarbonyl protecting group (Fmoc)/*tert*-butyl (tBu) solid-phase peptide strategy, purified and characterized by LC-MS (data not shown). In particular, five sequences have been generated, with two of them sharing a common chemical motif, proving effective in CCR4 antagonism and protection against *Aspergillus*, and now progressed to sequence optimization (data not shown).

In conclusion, the present manuscript demonstrates that CCR4 antagonists may be successfully used to prevent or treat aspergillosis in a variety of clinical settings and emphasizes the need for alternative drug development strategies for clinical translation.

## Data Availability

The data that support the findings of this study are available from the corresponding author upon reasonable request.

## References

[1] Griffith J.W., Sokol C.L., Luster A.D. (2014). Chemokines and chemokine receptors: Positioning cells for host defense and immunity. Annu. Rev. Immunol..

[2] Zlotnik A., Yoshie O. (2000). Chemokines: A new classification system and their role in immunity. Immunity.

[3] Dyer D.P. (2020). Understanding the mechanisms that facilitate specificity, not redundancy, of chemokine-mediated leukocyte recruitment. Immunology.

[4] Solari R., Pease J.E., Begg M. (2015). Chemokine receptors as therapeutic targets: Why aren’t there more drugs?. Eur. J. Pharmacol..

[5] Solari R., Pease J.E. (2015). Targeting chemokine receptors in disease—A case study of CCR4. Eur. J. Pharmacol..

[6] Hutchings C.J., Koglin M., Olson W.C., Marshall F.H. (2017). Opportunities for therapeutic antibodies directed at G-protein-coupled receptors. Nat. Rev. Drug Discov..

[7] Yoshie O., Matsushima K. (2015). CCR4 and its ligands: From bench to bedside. Int. Immunol..

[8] Santulli-Marotto S., Boakye K., Lacy E., Wu S.J., Luongo J., Kavalkovich K., Coelho A., Hogaboam C.M., Ryan M. (2013). Engagement of two distinct binding domains on CCL17 is required for signaling through CCR4 and establishment of localized inflammatory conditions in the lung. PLoS ONE.

[9] Marshall L.A., Marubayashi S., Jorapur A., Jacobson S., Zibinsky M., Robles O., Hu D.X., Jackson J.J., Pookot D., Sanchez J. (2020). Tumors establish resistance to immunotherapy by regulating Treg recruitment via CCR4. J. Immunother. Cancer.

[10] Ketcham J.M., Marshall L.A., Talay O. (2018). CCR4 Antagonists Inhibit Treg Trafficking into the Tumor Microenvironment. ACS Med. Chem. Lett..

[11] Romani L. (2011). Immunity to fungal infections. Nat. Rev. Immunol..

[12] Barnes P.D., Marr K.A. (2006). Aspergillosis: Spectrum of disease, diagnosis, and treatment. Infect. Dis. Clin. N. Am..

[13] Felton I.C., Simmonds N.J. (2014). Aspergillus and cystic fibrosis: Old disease—New classifications. Curr. Opin. Pulm. Med..

[14] Singh A., Ralhan A., Schwarz C., Hartl D., Hector A. (2018). Fungal Pathogens in CF Airways: Leave or Treat?. Mycopathologia.

[15] Burgel P.R., Paugam A., Hubert D., Martin C. (2016). Aspergillus fumigatus in the cystic fibrosis lung: Pros and cons of azole therapy. Infect. Drug Resist..

[16] Bell S.C., Mall M.A., Gutierrez H., Macek M., Madge S., Davies J.C., Burgel P.R., Tullis E., Castanos C., Castellani C. (2020). The future of cystic fibrosis care: A global perspective. Lancet Respir. Med..

[17] Costantini C., Puccetti M., Pariano M., Renga G., Stincardini C., D’Onofrio F., Bellet M.M., Cellini B., Giovagnoli S., Romani L. (2020). Selectively targeting key inflammatory pathways in cystic fibrosis. Eur. J. Med. Chem..

[18] van Doorninck J.H., French P.J., Verbeek E., Peters R.H., Morreau H., Bijman J., Scholte B.J. (1995). A mouse model for the cystic fibrosis delta F508 mutation. EMBO J..

[19] Bozza S., Clavaud C., Giovannini G., Fontaine T., Beauvais A., Sarfati J., D’Angelo C., Perruccio K., Bonifazi P., Zagarella S. (2009). Immune sensing of Aspergillus fumigatus proteins, glycolipids, and polysaccharides and the impact on Th immunity and vaccination. J. Immunol..

[20] Bayry J., Tchilian E.Z., Davies M.N., Forbes E.K., Draper S.J., Kaveri S.V., Hill A.V., Kazatchkine M.D., Beverley P.C., Flower D.R. (2008). In silico identified CCR4 antagonists target regulatory T cells and exert adjuvant activity in vaccination. Proc. Natl. Acad. Sci. USA.

[21] Davies M.N., Bayry J., Tchilian E.Z., Vani J., Shaila M.S., Forbes E.K., Draper S.J., Beverley P.C., Tough D.F., Flower D.R. (2009). Toward the discovery of vaccine adjuvants: Coupling in silico screening and in vitro analysis of antagonist binding to human and mouse CCR4 receptors. PLoS ONE.

[22] Montagnoli C., Fallarino F., Gaziano R., Bozza S., Bellocchio S., Zelante T., Kurup W.P., Pitzurra L., Puccetti P., Romani L. (2006). Immunity and tolerance to Aspergillus involve functionally distinct regulatory T cells and tryptophan catabolism. J. Immunol..

[23] Gregg K.S., Kauffman C.A. (2015). Invasive Aspergillosis: Epidemiology, Clinical Aspects, and Treatment. Semin. Respir. Crit. Care Med..

[24] Ridley C., Thornton D.J. (2018). Mucins: The frontline defence of the lung. Biochem. Soc. Trans..

[25] Mariani M., Lang R., Binda E., Panina-Bordignon P., D’Ambrosio D. (2004). Dominance of CCL22 over CCL17 in induction of chemokine receptor CCR4 desensitization and internalization on human Th2 cells. Eur. J. Immunol..

[26] Hartl D. (2009). Immunological mechanisms behind the cystic fibrosis-ABPA link. Med. Mycol..

[27] Schuh J.M., Power C., Proudfoot A.E., Kunkel S.L., Lukacs N.W., Hogaboam C.M. (2002). Airway hyperresponsiveness, but not airway remodeling, is attenuated during chronic pulmonary allergic responses to Aspergillus in CCR4-/- mice. FASEB J..

[28] Zhao J., Lloyd C.M., Noble A. (2013). Th17 responses in chronic allergic airway inflammation abrogate regulatory T-cell-mediated tolerance and contribute to airway remodeling. Mucosal Immunol..

[29] Iannitti R.G., Carvalho A., Cunha C., De Luca A., Giovannini G., Casagrande A., Zelante T., Vacca C., Fallarino F., Puccetti P. (2013). Th17/Treg imbalance in murine cystic fibrosis is linked to indoleamine 2,3-dioxygenase deficiency but corrected by kynurenines. Am. J. Respir. Crit. Care Med..

[30] Carpenter K.J., Hogaboam C.M. (2005). Immunosuppressive effects of CCL17 on pulmonary antifungal responses during pulmonary invasive aspergillosis. Infect. Immun..

[31] Pere H., Montier Y., Bayry J., Quintin-Colonna F., Merillon N., Dransart E., Badoual C., Gey A., Ravel P., Marcheteau E. (2011). A CCR4 antagonist combined with vaccines induces antigen-specific CD8+ T cells and tumor immunity against self antigens. Blood.

